# A new method of sentinel node mapping for early gastric cancer using a fluorescent laparoscope that can adjust the intensity of excitation light and quantify the intensity of indocyanine green fluorescence: Report of a case

**DOI:** 10.1016/j.ijscr.2020.07.045

**Published:** 2020-07-17

**Authors:** Teppei Kamada, Masashi Yoshida, Hideyuki Takeuchi, Satoshi Narihiro, Hironori Ohdaira, Yutaka Suzuki

**Affiliations:** Department of Surgery, International University of Health and Welfare Hospital, 537-3, Iguchi, Nasushiobara, Tochigi, 329-2763, Japan

**Keywords:** ICG, indocyanine green, SN, sentinel node, RGEA, right gastroepiploic artery, LGA, left gastric artery, SNNS, sentinel node navigation surgery, ICG, Sentinel node mapping, VISION SENSE

## Abstract

**Introduction:**

One of the drawbacks of indocyanine green (ICG) fluorescence sentinel node (SN) mapping is the impossibility of quantifying lymph node fluorescence during surgery. VISION SENSE® is a new near-infrared fluorescence laparoscope for bright-field full-color observation, and provides the ability to adjust the intensity of excitation light and quantify the intensity of ICG fluorescence during observation.

We report the case of a patient who underwent ICG SN mapping for early gastric cancer using VISION SENSE®.

**Presentation of case:**

A woman in her 60 s was diagnosed with cType0-IIc early gastric cancer located in the anterior wall of the middle gastric body (25 mm in diameter, cT1b, cN0, cM0, cStage IA). Contrast-enhanced computed tomography showed no metastases. Laparoscopy assisted distal gastrectomy with D1+ lymph node dissection and SN mapping with ICG fluorescence using the VISION SENSE® were successfully performed. Using VISION SENSE®, we could select those lymph nodes objectively showing high intensity by quantifying ICG fluorescence during surgery.

The pathological diagnosis was well-differentiated adenocarcinoma, pT1a, N0, M0, pStage IA. No ICG-positive lymph nodes (8 nodes) contained metastases.

Postoperative course was good, with no complications.

**Discussion:**

The use of VISION SENSE® allowing adjustment of excitation light and quantification of ICG fluorescence intensity might decreased the false-negative rate for SNs and increased the sensitivity of the ICG for detecting SNs.

**Conclusion:**

We successfully performed ICG SN mapping for early gastric cancer using VISION SENSE®.

## Highlights

• VISION SENSE® is a new near-infrared fluorescence laparoscope, and allows adjustment of the intensity of excitation light and quantification of the intensity of ICG fluorescence during observation.
• Using VISION SENSE®, when many ICG-positive lymph nodes are observed, we can select lymph nodes by quantifying ICG fluorescence during surgery.
• This procedure might improve SN mapping for early gastric cancer.

## 1 Introduction

Intensity of excitation light, sensitivity of fluorescent laparoscope and intra-lymphatic concentration of indocyanine green (ICG) are factors determining whether ICG fluorescence can be observed in sentinel node (SN) mapping for early gastric cancer.

One of the drawback of ICG fluorescence SN mapping is the impossibility of quantifying lymph node fluorescence during surgery.

VISION SENSE® (Medtronic, Minneapolis, MN, USA) is a new near-infrared fluorescence laparoscope for bright-field, full-color observation, and allows adjustment of the intensity of excitation light and quantification of the intensity of ICG fluorescence during observation. We hypothesized that SN mapping might be markedly change using VISION SENSE®, but a search of the literature revealed no reports on SN mapping using VISION SENSE®.

We report the case of a patient who underwent ICG SN mapping for early gastric cancer using VISION SENSE®.

The work has been reported in line with the SCARE criteria [1].

## 2 Presentation of case

A woman in her 60 s presented to our hospital after an abnormality was detected on upper gastrointestinal series. She was diagnosed with cType0-IIc early gastric cancer located in the anterior wall of the middle gastric body (25 mm in diameter, cT1b, cN0, cM0, cStage IA).

Results of blood examination on admission were unremarkable. Contrast-enhanced computed tomography showed no metastases.

Laparoscopy assisted distal gastrectomy with D1+ lymph node dissection was planned according to the criteria for gastric cancer treatment guidelines of the Japanese Gastric Cancer Association (JGCA). SN mapping using an ICG fluorescence method was performed.

The protocol for the ICG fluorescence method was approved by the Ethics Committee for Biomedical Research of our hospital, and the patient provided written informed consent (Approval No. 13-B-60).

### 2.1 Surgical procedure

**Fig. 1:**
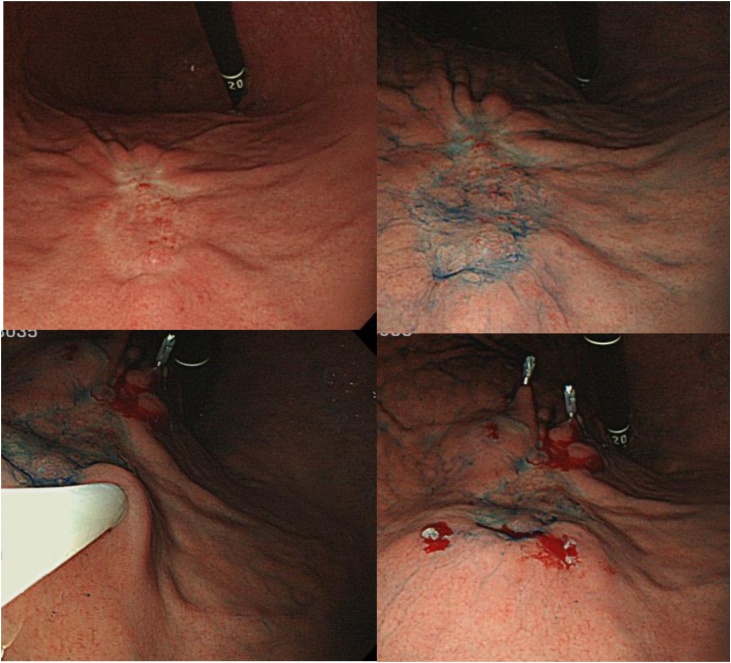
The day before surgery, 0.5 mL of 50.0 μg/mL ICG was injected into each of four quadrants of the submucosal layer of the primary lesion using an endoscopic puncture needle.

Injection of the tracer was performed as Ohdaira et al. reported [2]. The day before surgery, ICG (Diagnogreen®; Dai-Ichi Sankyo Pharm, Tokyo, Japan) was injected into four quadrants of the submucosal layer of the tumor using an endoscopic puncture needle (Fig. 1). Each quadrant received 0.5 mL of 50.0-μg/mL ICG.

**Fig. 2:**
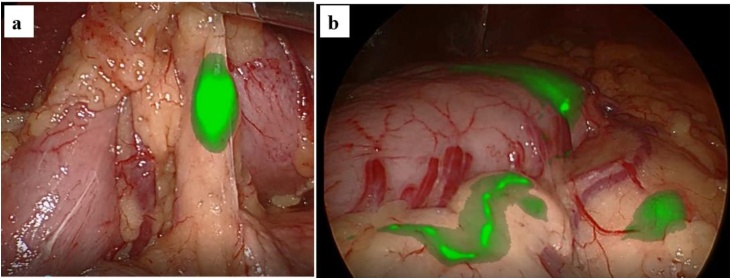
ICG-positive lymphatic flow and lymph nodes are observed in the areas of the right gastroepiploic artery (RGEA) and left gastric artery (LGA).LGA area (b) RGEA area.

VISION SENSE® was used for observation of ICG fluorescence. ICG-positive lymphatic vessels and lymph nodes were able to be observed in the areas of the right gastroepiploic artery (RGEA) and left gastric artery (LGA) (Fig. 2a, b). Laparoscopy assisted distal gastrectomy with D1+ lymph node dissection was then performed.

**Fig. 3:**
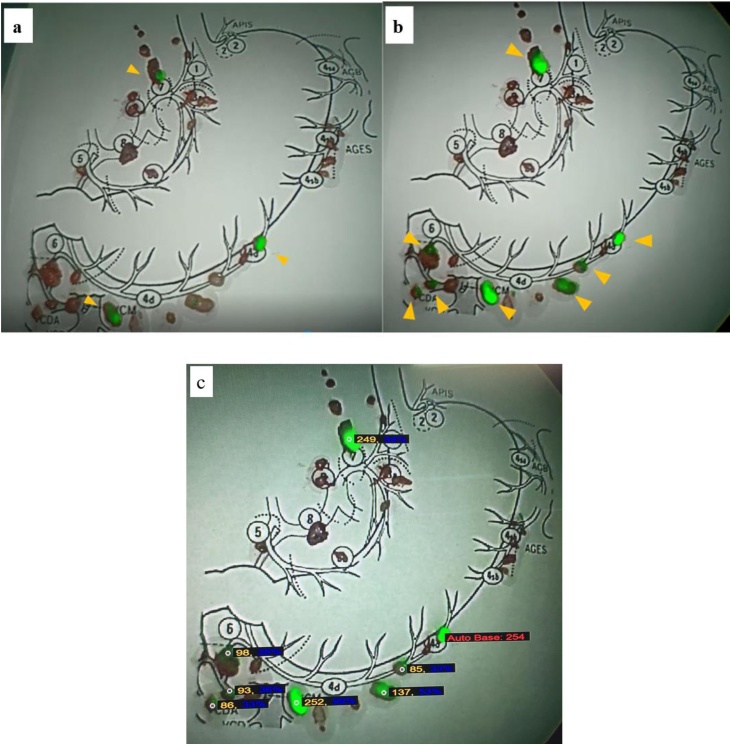
Back-table observation of bright lymph nodes (a) Three ICG-positive lymph nodes (arrowhead) are visible under low-IR boost observation. (b) Eight ICG-positive lymph nodes (arrowhead) are visible under high-IR boost observation. (c) Quantification of the intensity of ICG fluorescence (red: baseline; autobase: 254). Maximum intensity of ICG fluorescence is marked as autobase, and the intenstity of ICG fluorescence compared to autobase is marked (yellow: measured value; blue: percentage).

Postoperatively, back-table observation of bright lymph nodes and pathological examination with hematoxylin and eosin staining was performed. Three ICG-positive lymph nodes were observed under weak excitation light (Fig. 3a). Eight ICG-positive lymph nodes were observed as the intensity of excitation light was increased (Fig. 3b). Intensity of ICG fluorescence could be quantified, confirming a difference in ICG fluorescence intensity of the bright lymph node during surgery (Fig. 3c).

The pathological diagnosis was well-differentiated adenocarcinoma, pT1a, N0, M0, pStage IA. No ICG-positive lymph nodes (8 nodes) contained metastases.

Postoperative course was good, with no complications.

## 3 Discussion

The number of ICG-positive lymph nodes changed as we adjusted the intensity of excitation light using VISION SENSE®, which allows adjustment of the intensity of excitation light and quantification of ICG fluorescence intensity. These properties might allow improved SN mapping for early gastric cancer. When only a small number of ICG-positive lymph nodes can be observed, a greater number of ICG-positive lymph nodes can be observed by increasing the intensity of excitation light. When many ICG-positive lymph nodes can be observed, we can select the most appropriate lymph nodes by quantifying ICG fluorescence.

An understanding of the concept and history of past SN mapping is necessary to discuss the number of SNs in gastric cancer. Gastrectomy with D2 lymph node dissection is currently regarded as a standard approach for gastric cancer, even in some patients with early gastric cancer. However, gastrectomy with D2 lymph node dissection is associated with the development of post-gastrectomy syndrome. SN navigation surgery (SNNS) has thus been expected to provide a useful option in optimizing the balance between post-surgical quality of life and curability in the clinical management of patients with early gastric cancer.

SNNS is considered useful for gastric cancer showing abundant and complicated lymph flow and high possibility of skip metastasis, because it facilitates recognition of the dynamics of the lymphatic vessels and basin [3,4].

Many studies have reported on the feasibility of SN mapping for gastric cancer, including two epoch-making prospective multicenter trials from Japan. Kitagawa et al. [5] suggested SN mapping with the dual-tracer method (using Technetium 99 m tin colloid solution (0.5 mL × 4 points) and 1% isosulfan blue dye) as a feasible and safe procedure for gastric cancer, offering 99% accuracy of nodal evaluation for metastasis. On the basis of that study, a multicenter trial was conducted to evaluate the feasibility and accuracy of SN-navigated modified gastrectomy for T1 gastric cancer (<4 cm, single tumor). The other multicenter trial (JCOG0302) included SN biopsy with dye method (ICG without fluorescent scopes) as a single tracer, but that trial was discontinued because of an unacceptably high false-negative rate (46%). The median number of ICG-positive lymph nodes in that study was 4 [6]. The dual-tracer method has thus been considered the standard of SN mapping in gastric cancer. However, the dual-tracer method requires endoscopic injection of radioactive colloid in a radiation controlled area. ICG fluorescence laparoscopic surgery is a simpler method that does not need an endoscope always ready in the radiation controlled area.

In 2004, Nimura et al. first reported infrared-guided surgery with ICG for detecting SNs in patients with gastric cancer [7]. In 2008, Kusano et al. reported the examination of 22 patients with gastric cancer and 26 patients with colorectal cancer by detecting SNs with ICG fluorescence imaging using a charge-coupled device. The mean number of SNs identified was 3.6 ± 4.5, with 88.9% accuracy [8].

Many devices are available for ICG fluorescence-guided methods of SN mapping. Kinami et al. applied the Photodynamic Eye® (PDE; Hamamatsu Photonics Co., Shizuoka, Japan) to identify ICG fluorescence [9]. The median number of SNs identified was 6. That study of 72 patients showed equivalent accuracy to the dual-tracer method.

Yoshida et al. applied the Hyper Eye Medical System® (HEMS; Mizuho Corporation, Tokyo, Japan) for ICG fluorescence SNs mapping [10]. In that study, bright-field observation under room light facilitated selection of bright nodes, as non-bright nodes could not be observed under room light.

Takahashi et al. applied the infrared ray laparoscopic system (IRLS; Olympus Optical, Tokyo, Japan) for early gastric cancer surgery. In that study of 44 patients, 7 cases showed lymph node metastases, and the false-negative rate was 0%. The mean number of identified SNs was 7.9 [11].

Ohdaira et al. applied PINPOINT® (Stryker, Kalamazoo, MI, USA) for ICG fluorescence SN mapping (bright-field, full-color observation) [2]. The mean number of SNs identified was 8.6.

Furthermore, Okubo et al. reported that evaluation of fluorescence intensity using ICG intensity imaging software® (Mizuho Corporation, Tokyo, Japan) after surgery was useful for selected SNs. Dissection of 6 ICG fluorescent nodes by evaluating levels of fluorescence intensity with ICG intensity imaging software® after surgery included 92.1% of SNs selected by the RI method [12].

We therefore considered that regardless of administration concentrations and timing of tracer injection, we should detect at least an average of 6–8 lymph nodes from lymph nodes showing high fluorescence intensity. This is because the mean number of SNs identified was 5.6 in prospective multicenter trials with the dual-tracer method [5], and recent ICG fluorescence studies [2,9,11,12] with acceptable results have shown equivalent number of SNs (>6), while ≤4 SNs were obtained in trials with high false-negative rates.

Identifying the appropriate number of SNs is desirable. Frequent identification and collection of more than 10 SNs is unsuitable for frozen section diagnosis because of the burden on pathologists and the long diagnosis time. We therefore applied VISION SENSE® for SN mapping. Using VISION SENSE®, when only a few ICG-positive lymph nodes can be observed, a greater number of ICG-positive lymph nodes can be observed by increasing the intensity of the excitation light. When many ICG-positive lymph nodes can be observed, we can select those lymph nodes showing the highest intensity by objectively quantifying the ICG fluorescence during surgery.

Results from this case appear good enough to initiate further study and accumulation of cases. The use of VISION SENSE® allowing adjustment of excitation light and quantification of ICG fluorescence intensity may decreased the false-negative rate for SNs and increased the sensitivity of the ICG for detecting SNs.

## 4 Conclusion

We report the case of a patient who underwent ICG SN mapping for early gastric cancer using VISION SENSE®. This procedure might improve SN mapping for early gastric cancer.

## Conflicts of interest

There are no conflicts of interest.

## Funding

We have no sponsors.

## Ethical approval

This study was approved (Approval No. Approval No. 13-B-60) by the Clinical Ethics Committee of International University of Health and Welfare, Tochigi, Japan.

## Consent

Written informed consent was obtained from the patient for publication of this case report and accompanying images. A copy of the written consent is available for review by the Editor-in-Chief of this journal on request”

## Author contribution

TK: study design, data collection, data analysis, writing.

MY: critical revision.

YS: final approval of the article.

Any other authors: data collection.

All authors read and approved the final manuscript.

## Registration of research studies

This paper is case report. The authors don’t need to register this work.

## Guarantor

Teppei Kamada, the corresponding author of this manuscript accept full responsibility for the work and the conduct of the study, access to the data and controlled the decision to publish.

## Provenance and peer review

Not commissioned, externally peer-reviewed.
